# MRI index lesion radiomics and machine learning for detection of extraprostatic extension of disease: a multicenter study

**DOI:** 10.1007/s00330-021-07856-3

**Published:** 2021-04-01

**Authors:** Renato Cuocolo, Arnaldo Stanzione, Riccardo Faletti, Marco Gatti, Giorgio Calleris, Alberto Fornari, Francesco Gentile, Aurelio Motta, Serena Dell’Aversana, Massimiliano Creta, Nicola Longo, Paolo Gontero, Stefano Cirillo, Paolo Fonio, Massimo Imbriaco

**Affiliations:** 1 Department of Clinical Medicine and Surgery, University of Naples “Federico II”, Naples, Italy; 2 Laboratory of Augmented Reality for Health Monitoring (ARHeMLab), Department of Electrical Engineering and Information Technology, University of Naples “Federico II”, Naples, Italy; 3 Department of Advanced Biomedical Sciences, University of Naples “Federico II”, Naples, Italy; 4 Department of Surgical Sciences, Radiology Unit, University of Turin, Via Genova 3, 10126 Turin, Italy; 5 Division of Urology, Città della Salute e della Scienza, Molinette Hospital, University of Turin, Torino, Italy; 6 Radiology Unit, Mauriziano Umberto I Hospital, 10128 Turin, Italy; 7 Department of Neurosciences, Reproductive Sciences and Odontostomatology, University of Naples “Federico II”, Naples, Italy

**Keywords:** Magnetic resonance imaging, Machine learning, Support vector machine, Prostate cancer, Prostatectomy

## Abstract

**Objectives:**

To build a machine learning (ML) model to detect extraprostatic extension (EPE) of prostate cancer (PCa), based on radiomics features extracted from prostate MRI index lesions.

**Methods:**

Consecutive MRI exams of patients undergoing radical prostatectomy for PCa were retrospectively collected from three institutions. Axial T2-weighted and apparent diffusion coefficient map images were annotated to obtain index lesion volumes of interest for radiomics feature extraction. Data from one institution was used for training, feature selection (using reproducibility, variance and pairwise correlation analyses, and a correlation-based subset evaluator), and tuning of a support vector machine (SVM) algorithm, with stratified 10-fold cross-validation. The model was tested on the two remaining institutions’ data and compared with a baseline reference and expert radiologist assessment of EPE.

**Results:**

In total, 193 patients were included. From an initial dataset of 2436 features, 2287 were excluded due to either poor stability, low variance, or high collinearity. Among the remaining, 14 features were used to train the ML model, which reached an overall accuracy of 83% in the training set. In the two external test sets, the SVM achieved an accuracy of 79% and 74% respectively, not statistically different from that of the radiologist (81–83%, *p* = 0.39–1) and outperforming the baseline reference (*p* = 0.001–0.02).

**Conclusions:**

A ML model solely based on radiomics features demonstrated high accuracy for EPE detection and good generalizability in a multicenter setting. Paired to qualitative EPE assessment, this approach could aid radiologists in this challenging task.

**Key Points:**

*• Predicting the presence of EPE in prostate cancer patients is a challenging task for radiologists.*

*• A support vector machine algorithm achieved high diagnostic accuracy for EPE detection, with good generalizability when tested on multiple external datasets.*

*• The performance of the algorithm was not significantly different from that of an experienced radiologist.*

**Supplementary Information:**

The online version contains supplementary material available at 10.1007/s00330-021-07856-3.

## Introduction

The diagnostic pathway for prostate cancer (PCa) is rapidly evolving, with multiparametric MRI (mpMRI) gaining an increasingly central role in tumor detection. Indeed, it allows to identify lesions worthy of targeted biopsies, which when paired to systematic sampling leads to a more accurate and clinically relevant PCa assessment [1–3]. However, the value of mpMRI could go beyond PCa detection, and a great attention is presently paid to its accuracy in the identification of extraprostatic extension of disease (EPE) [4]. While confirmed organ-confined disease at mpMRI could lead to more conservative surgical approaches, mpMRI suffers from a relatively low and heterogeneous sensitivity that currently prevents its widespread adoption for this task [5, 6]. This could be at least in part due to a lack of standardization in the interpretation of the multiple EPE signs detectable at mpMRI, although inherent limitations of the technique cannot be excluded. In this light, strategies to increase the performance of mpMRI for PCa local staging have been recently investigated and dedicated scoring systems for the reporting of EPE on mpMRI have been released, with the one named EPE grade proposed by Mehralivand et al that appears to be the most promising and awaiting validation [7–10]. Among the several mpMRI features suspicious for EPE, this scoring system focuses on tumor capsular contact length, capsular bulge, and/or irregularity and frank capsular breach [9]. However, there are other recognized mpMRI signs suggested by the latest release of Prostate Imaging and Reporting Data System (PI-RADS v2.1) that radiologists should consider in their decision making regarding EPE prediction, such as asymmetry of the neurovascular bundles and obliteration of the rectoprostatic angle [11]. Furthermore, the optimal tumor capsular contact threshold for EPE prediction still needs to be defined with EPE grade and PI-RADS v2.1 suggesting different cut-offs [9, 11–13]. Concurrently, prostate mpMRI has been one of the many imaging modalities’ object of study in the field of radiomics, which is a multi-step process allowing the extraction of quantitative features from medical images that can be used to build decision support models [14–16]. Exploratory radiomics studies have been performed to assess the feasibility of applying radiomics, in combination with machine learning (ML) or not, for EPE prediction on mpMRI images, with encouraging results [17–21]. Nevertheless, their findings were limited in terms of generalizability due to the adoption of single-center datasets. With the present work, we aimed to evaluate the performance of a ML algorithm powered by radiomics data alone in the identification of EPE and compare its diagnostic accuracy to that of expert radiologists, using two independent external datasets for validation.

## Materials and methods

The respective Local Institutional Review Board for each Institution approved this retrospective study and waived the need for written informed consent.

### Patient population

This study was conducted enrolling patients from Molinette Hospital, Turin (site 1); Mauriziano Umberto I Hospital, Turin (site 2); and Federico II Hospital, Naples (site 3), Italy. We retrospectively reviewed consecutive patients who underwent prostate MRI at each site for PCa suspicion between November 2015 and October 2018. Inclusion criteria were the following: presence of an index lesion (PI-RADS score ≥ 3, defined according to PI-RADS guidelines, assigned by the original reader) with bioptic confirmation of PCa presence (defined as Gleason score ≥ 3 + 3) in the index lesion through targeted biopsy within 3 months of MRI; treatment with RP within 3 months of biopsy. Only patients with significant artifacts at MRI or incomplete exams (i.e., interrupted for claustrophobia) were excluded from subsequent analyses. Pathology reports were analyzed to assess if EPE was identified on RP specimens at the location of the index lesion, based on the International Society of Urological Pathology consensus conference criteria [22]. Patients from site 1 were used to train and tune the ML model while those from sites 2 and 3 were employed as distinct external test sets to validate its performance.

### MRI acquisition

MRI examinations at sites 1 and 2 were performed on 1.5-T scanners (Achieva and Ingenia, Philips Medical Systems). A 3-T scanner was employed in site 3 (Magnetom Trio, Siemens Medical Solutions), using surface phase-arrayed and integrated spine phased-array coils. None of the sites employed endorectal coils. All acquisition protocols included axial T2-weighted (T2w) and diffusion-weighted images (DWI), with corresponding apparent diffusion coefficient maps (ADC). Further details are available in the supplementary materials.

### Radiomics analysis

**Fig. 1:**
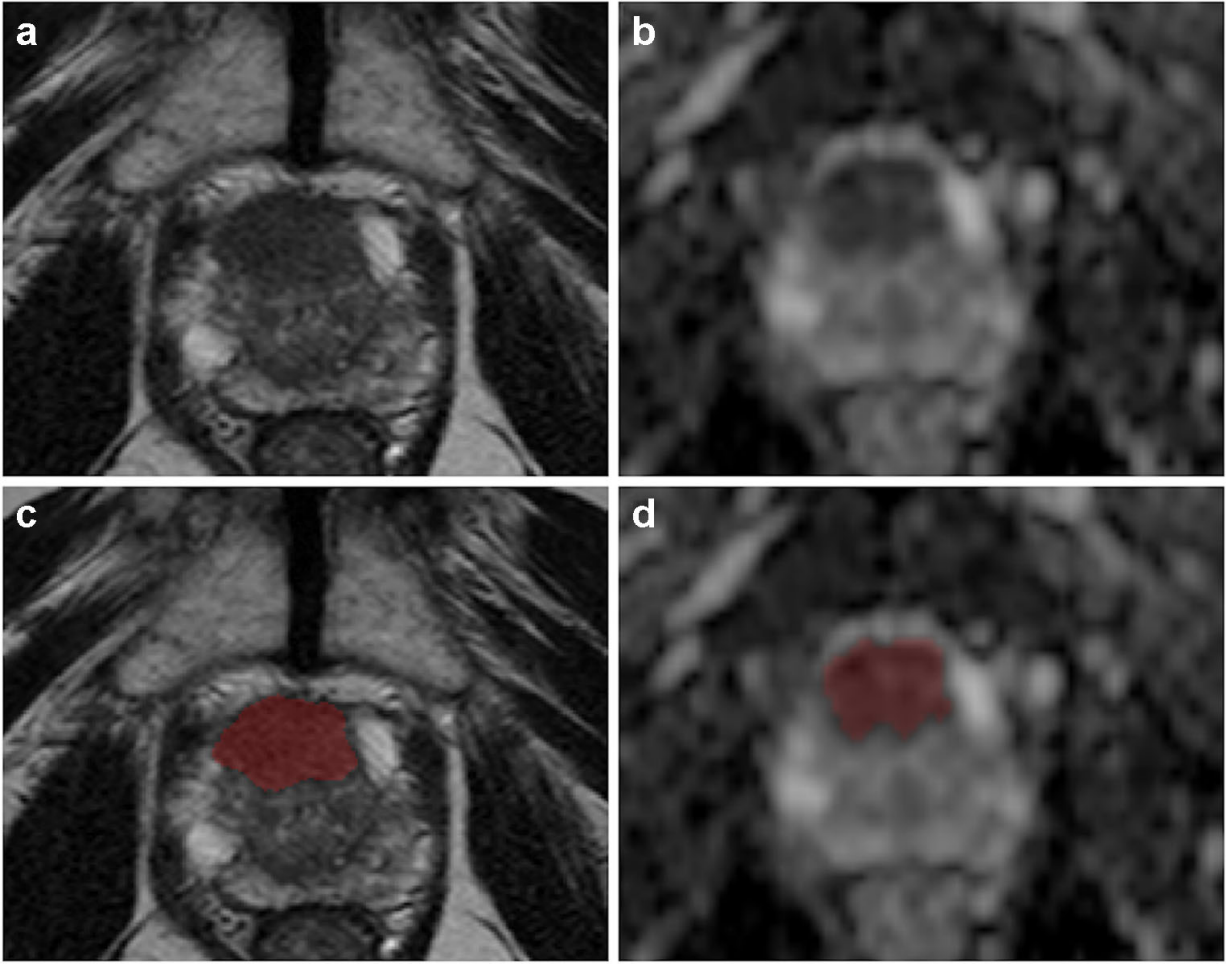
Prostate MR images (axial T2-weighted on the left and ADC map on the right) from a 76-year-old patient with a PI-RADS 5 transition zone lesion involving the anterior fibromuscular stroma (Gleason score 4+3 and signs of extraprostatic extension of disease at prostatectomy). The slices in which the lesion was more conspicuous are shown respectively before (**a** and **b**) and after (**c** and **d**) after manual segmentation

All axial T2w and ADC images from included patients were manually anonymized and converted to the NIfTI format prior to analysis, using dcm2niix [23]. Index lesion location was provided to a genitourinary radiologist (> 5 years of experience), who performed a manual segmentation of the entire index lesion volume on both T2w and ADC images (Fig. 1). To assess the feature reproducibility in relation to manual segmentation, two other readers (a radiologist and a radiology resident) independently annotated a subset of 30 randomly selected patients from the site 1 training set. The supplementary materials contain further details on the additional annotations. These segmentations were used for intercorrelation coefficient (ICC) calculation in one of the feature selection steps as detailed below. Dedicated software was used for all segmentations (ITK-SNAP, v3.8) [24].

PyRadiomics (v3.0) was employed for feature extraction [25]. Preprocessing was performed with voxel resampling to 1 × 1 × 1 mm, whole-image gray level *z*-score normalization, scaling by a 100 factor and array shift (by 300), followed by discretization with a fixed bin width (= 5). Both original and filtered images were used to calculate 3D shape, first order, and texture features. In detail, the Laplacian of Gaussian filtering with multiple sigma values (= 1, 2, 3, 4, 5) and wavelet decomposition with all combinations of high- and low-pass filters in the *x-*, *y-*, and *z*-axes were employed to highlight textural characteristics of the index lesion. The supplementary materials contain the complete settings file used for the extraction.

### Feature preprocessing and selection

The scikit-learn Python3 package and the Weka data mining software were used for the subsequent steps of the analysis (v3.8) [26, 27]. The feature selection process was conducted exclusively on the site 1 training dataset, to avoid any information leak which could bias the final model. A normalization scaler (range = 0–1) was fit on the training data and used to transform all 3 datasets.

The first step of feature selection consisted in the exclusion of non-reproducible features through ICC analysis of the results obtained by the three independent readers. Radiomics features were extracted, using the same settings, from their respective annotation sets (*n* = 30). The resulting datasets were used to calculate the ICC value of each parameter. A two-way random effect, single rater, absolute agreement ICC model was employed, and a value ≥ 0.75 was considered “good reproducibility,” the minimum requirement for inclusion in the analysis [28]. Subsequently, a variance filter (threshold = 0.1) was applied to each feature to remove parameters with low information content. Using pairwise correlation, features with high collinearity (threshold > 0.8) were also excluded from the analysis. Finally, the Weka data mining platform (v3.9) correlation-based feature subset evaluator was used to identify the best feature subset among the remaining.

### Machine learning

A support vector machine (SVM) algorithm was employed to develop a predictive model for EPE. Training set classes were balanced using the Synthetic Minority Oversampling Technique [29]. A stratified 10-fold cross-validation was used for model tuning prior to final training on the entire site 1 dataset. The final model performance was then independently tested on 2 external datasets (sites 2 and 3) calculating confusion matrix--derived accuracy metrics and receiver operating characteristics (ROC) curves. Brier score and calibration curves were also obtained for each test set to evaluate prediction and calibration loss. For each center included in the study, an expert radiologist (all > 5 years of experience in prostate MRI) performed an assessment based on the entire prostate mpMRI exam and each case was classified as positive or negative for EPE using previously established signs from the PI-RADSv2.1 guidelines [30]. No PI-RADS scores were assigned during these readings since PI-RADS scores were not included in the ML analysis. These readings were used to provide a comparison for the SVM’s performance.

**Fig. 2:**
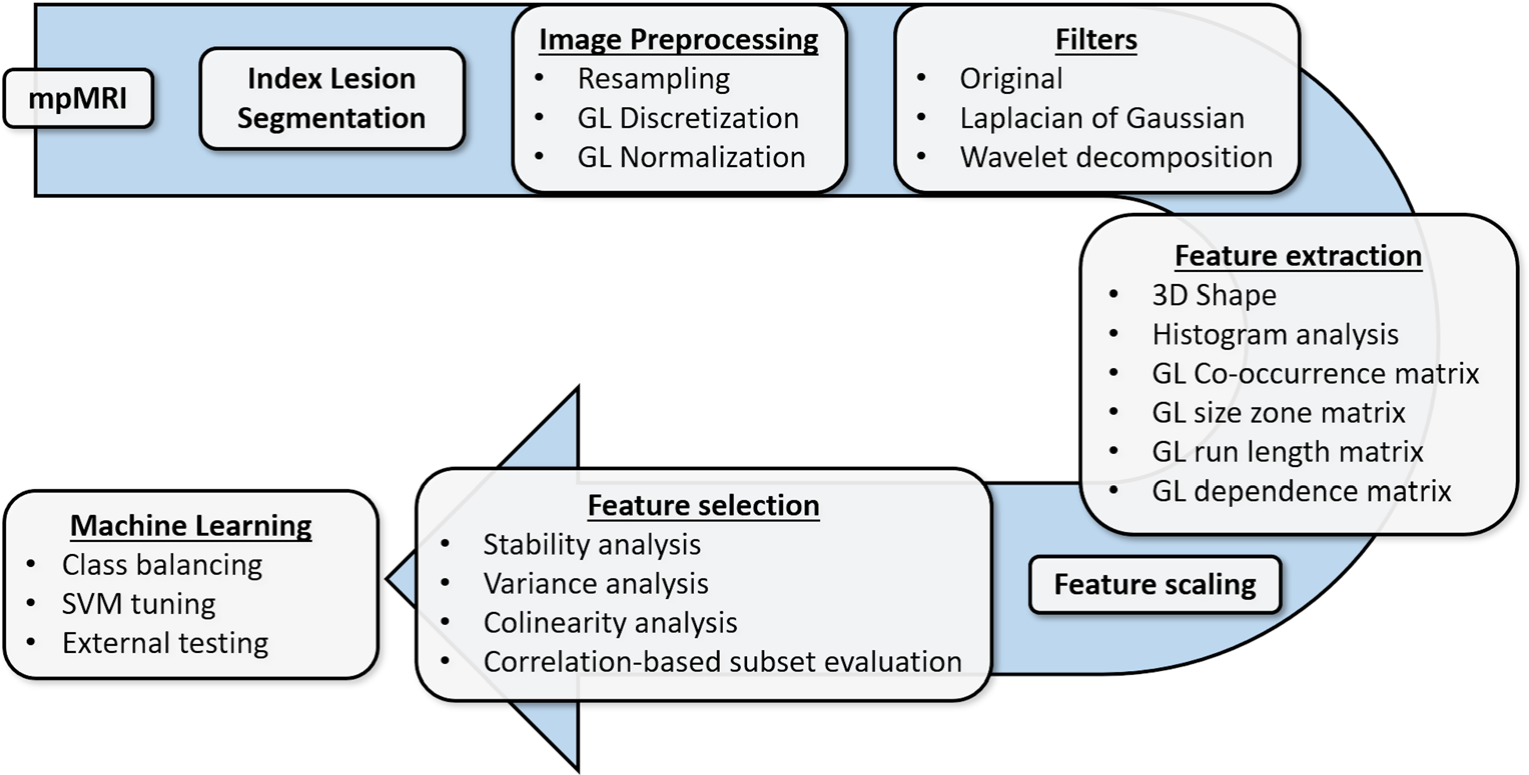
Image analysis and machine learning pipeline

An overview of the complete analysis pipeline is presented in Fig. 2.

### Statistical analysis

Continuous variables were tested for normality using the Shapiro-Wilk test and are presented as mean and standard deviation or median and interquartile range accordingly. Ordinal data are presented as value counts, categorical data as proportions. The Kruskal-Wallis rank sum test was used to assess differences in clinical data among the datasets, with a Dunn test post hoc analysis if necessary. A Fisher exact test was used to compare the distribution of EPE cases between the 3 sites. Model accuracy (*n* correct predictions/total cases) in each test set was also compared with a baseline reference (no information rate, i.e., the class mode) using a binomial test and an expert radiologist’s predictions using McNemar’s tests. A *p* < 0.05 was considered statistically significant, with correction for multiple comparisons when required. The statistical analysis was performed using the R software environment [31].

## Results

**Fig. 3:**
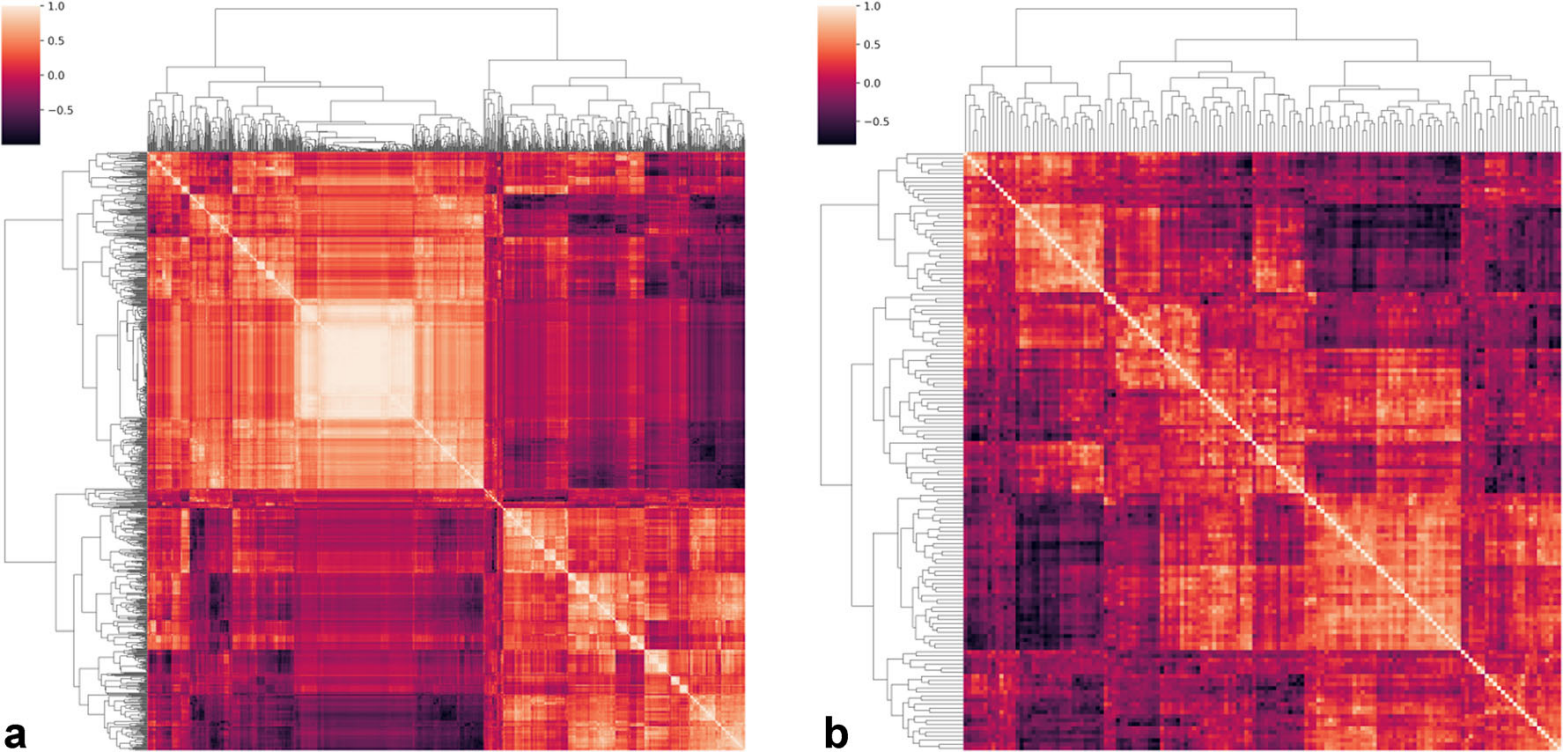
Hierarchically clustered heatmap of feature pairwise correlation before (**a**) and after (**b**) removal of highly colinear ones

**Fig. 4:**
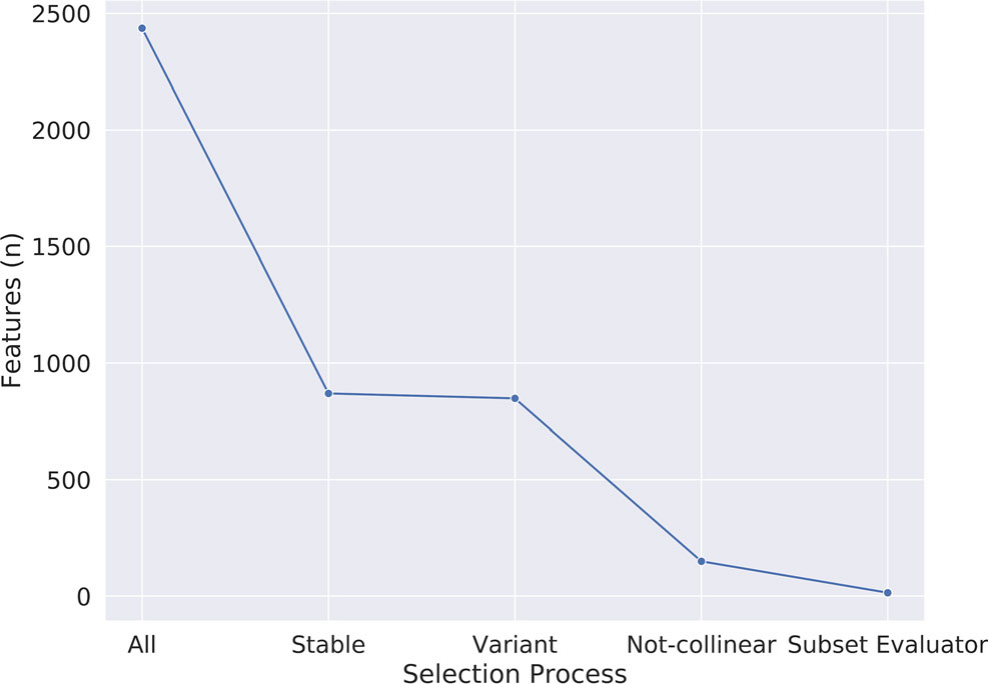
Plot depicting parameter number (*y*-axis) reduction during the various feature selection steps (*x*-axis)

In total, 193 patients met the selection criteria: 104 from site 1, 43 from site 2, and 46 from Site 3. Their clinical and demographic data are reported in Table 1. No significant differences were found among the three populations, with the exception of PI-RADS scores of Sites 2 and 3 (*p* = 0.008). From their exams, 1218 radiomics features were extracted from T2w and ADC images (complete dataset = 2436 features). Of these, 55% (*n* = 675/1218) from T2w images and 73% (*n* = 892/1218) from ADC maps resulted not reproducible. Among the remaining 869 parameters, 21 (2%) presented low variance and were also removed from the training dataset. The intercorrelation analysis led to the exclusion of an additional 699/848 features (82%) and the correlation-based subset evaluator identified 14 features to be employed in training the model, listed in the supplementary materials (Figs. 3 and 4).

**Table 1:** Patient population clinical and demographic characteristics. Continuous data are presented as a median and interquartile range, ordinal data as value counts, and categorical data as proportions

	Site 1	Site 2	Site 3	
Age (years)	66 (60–72)	67 (60–69)	67 (63–71)	*p = 0.32*
PSA (ng/ml)	7.1 (5.12–10.00)	6.93 (5.51–9.78)	8.00 (5.35-9.76)	*p = 0.89*
ISUP grade^§^	1 = 1	1 = 1	1 = 5	*p = 0.21*
	2 = 40	2 = 21	2 = 15	
	3 = 43	3 = 16	3 = 8	
	4 = 17	4 = 4	4 = 14	
	5 = 3	5 = 1	5 = 5	
PI-RADS score^#^	3 = 3	3 = 7	3 = 3	p = 0.02*
	4 = 68	4 = 24	4 = 19	
	5 = 33	5 = 12	5 = 25	
EPE (pathologically proven)	37/104	19/43	20/47	*p = 0.55*

*PSA*, prostate-specific antigen; *ISUP*, International Society of Urological Pathology; *PI-RADS*, Prostate Imaging and Reporting Data System; *EPE*, extraprostatic extension of disease

*Post hoc analysis showed a significant difference exclusively between sites 2 and 3 (*p = 0.008*)

^§^As originally assigned by the pathologist on the radical prostatectomy specimen

^#^Obtained from the original radiology report

**Fig. 5:**
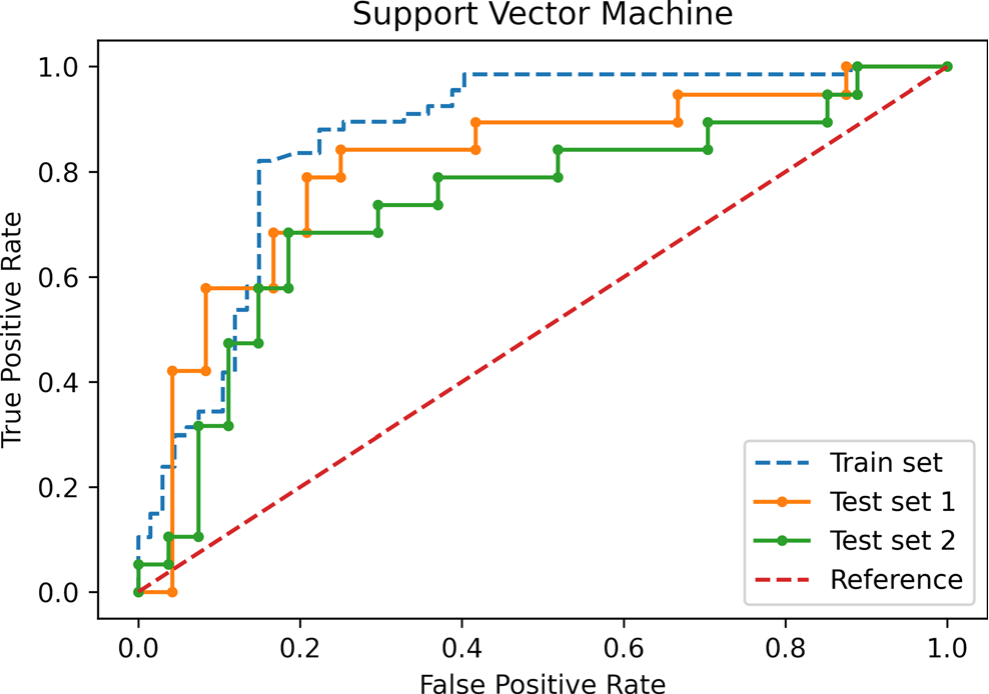
Receiver operating characteristic curves of the support vector machine model in the train data and both test sets

**Fig. 6:**
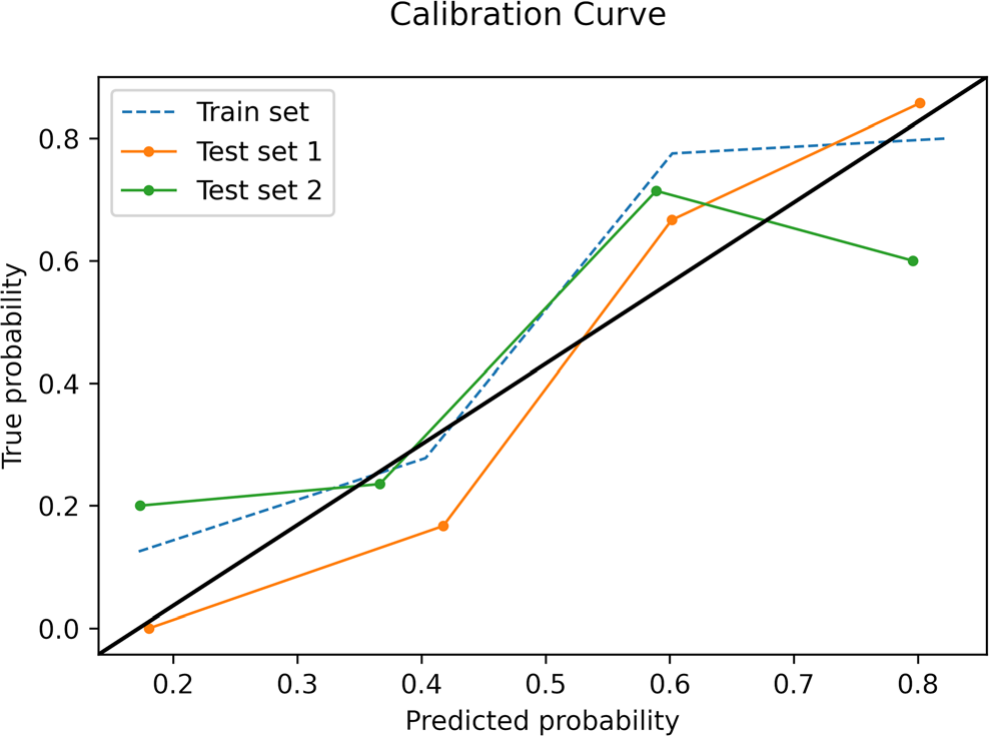
Calibration curves of the support vector machine model in the train data and both test sets

The final model was an SVM classifier with a radial basis function kernel, *C* = 3 and gamma = 0.01; the trained model file is available in the supplementary materials. In the training set, an overall accuracy (correct/overall instances) of 83% was obtained, with a 0.83 area under the ROC curve (AUC). In the external test sets, the SVM reached an accuracy of 79% and AUC of 0.80 in the data from site 2 and 74% and 0.73 in site 3 (Fig. 5). The Brier score was 0.20 and 0.21 for sites 2 and 3 data, respectively (Fig. 6). In both test sets, ML outperformed the baseline reference (*p* = 0.001 in site 2, *p* = 0.02 in site 3). Confusion matrices and complete accuracy metrics are reported in Tables 2 and 3, respectively. The radiologist achieved an accuracy of 81% and 83% respectively in sites 2 and 3. Both did not reach statistical significance when compared to ML (*p* = 1 for site 2 and *p* = 0.39 for site 3) (Table 4). The confusion matrices for the radiologist’s assessment are presented in the supplementary materials.

**Table 2:** Confusion matrices for the SVM model

Sites		Ground truth	
		No EPE	EPE
Site 2			
SVM	No EPE	18	3
	EPE	6	16
Site 3			
SVM	No EPE	21	6
	EPE	6	13

*SVM*, support vector machine; *EPE*, extraprostatic extension of disease

**Table 3:** Accuracy metrics for the SVM model for the training (site 1) and testing (site 2 and site 3) datasets

	Sensitivity	Positive predictive value	F-measure	MCC	AUC	AUPRC
Site 1						
Absence of EPE	0.78	0.87	0.82	0.66	0.83	0.79
Presence of EPE	0.88	0.80	0.84	0.66	0.83	0.76
Weighted Average	0.83	0.83	0.83	0.66	0.83	0.77
Site 2						
Absence of EPE	0.75	0.86	0.80	0.59	0.80	0.78
Presence of EPE	0.84	0.73	0.78	0.59	0.80	0.68
Weighted Average	0.79	0.80	0.79	0.59	0.80	0.74
Site 3						
Absence of EPE	0.78	0.78	0.78	0.46	0.73	0.74
Presence of EPE	0.68	0.68	0.68	0.46	0.73	0.60
Weighted Average	0.74	0.74	0.74	0.46	0.73	0.68

*EPE*, extraprostatic extension of disease; *MCC*, Matthew’s correlation coefficient; *AUC*, area under the receiver operating characteristics curve; *AUPRC*, area under the precision-recall curve

**Table 4:** Comparison of the SVM model and radiologist performances for the McNemar test

Sites		SVM	
		Error	Correct
Site 2			
Radiologist	Error	4	4
	Correct	5	30
Site 3			
Radiologist	Error	4	4
	Correct	8	30

*SVM*, support vector machine

## Discussion

Our study demonstrates the potential of radiomics-powered ML for the detection of EPE in PCa, which in turn could improve patient management and treatment choice. There have been several prior investigations that have focused on this task using a similar approach [17–21]. In our previous, single-center exploratory study of radiomics and ML for EPE detection, the accuracy achieved was similar (82%), although only obtained through cross-validation in a single dataset [21]. In a prior investigation, Krishna et al had identified ADC map first-order features, in particular entropy, as promising EPE biomarkers (AUC 0.76) [19]. More recently, Ma et al obtained an 83% accuracy in a single institution 3T dataset, using 67 patients for testing the model [20]. Interestingly, in their study, they reported that the radiomics approach significantly outperformed the radiologist in EPE assessment, but exclusively in terms of sensitivity and not specificity or overall accuracy. Losnegard et al analyzed data acquired on a 1.5-T scanner using an endorectal coil, and their random forest model achieved an AUC of 0.74 [17]. This was again very similar to that of the radiologist (0.75) on the same data. Finally, Xu et al were able to obtain an 82% accuracy on their test set, using data acquired on a 3-T scanner [18]. Our results in the training dataset are essentially in line with these previous studies (83% accuracy; however, none of these has performed external testing.

In the present investigation, we decided not to employ dynamic contrast-enhanced images (DCE) to obtain radiomics data on which to build our predictive model. This choice was dictated by several considerations, mainly the concern to ensure the widest possible applicability and generalizability of the resulting model as well as reducing sources of variability in our data as much as reasonably possible. Regarding the first, biparametric prostate MRI protocols without the use of DCE are becoming more and more common, also to accommodate the increasing demand for MRI exams due to its growing role in current guidelines [5, 32]. Protocols without DCE have also shown a similar performance to full mpMRI for EPE detection [33]. Finally, DCE has a high temporal resolution, with the degree and speed of lesion enhancement influenced both by technical and physiological factors, together with lesion nature. Therefore, it would be challenging to exclude all sources of bias from DCE radiomics features, potentially adding more noise to the data. Therefore, in the interest of keeping the model as simple as possible and employable in most clinical settings, we decided to only focus on T2w and ADC features for EPE predictive modeling.

As reproducibility of results represents one of the main limitations of both radiomics and ML, we chose a multicentric design to have the possibility to directly assess this issue by testing our model’s results generalizability [34, 35]. It should also be noted that our external test sets were constituted by exams acquired on different scanners with varying field strength compared to the training data. Our intention was to offer a better representation of real-world clinical practice and a better estimation of our model’s performance. The results are very promising, with an accuracy of 83% in the training and 79% and 74% in the test sets and a performance comparable to that of an expert radiologist in both cases (*p* = 1.00 and 0.39, respectively). It can be assumed that image preprocessing paired with robust feature selection, including feature stability testing, has reduced overfitting on noisy data. However, it should be noted that performance on the site 3 test set was still somewhat lower (74%) than on site 2 (79%). A bias due to case sampling cannot be completely excluded; i.e., more challenging cases were randomly present in one test set compared to the other, and this could be supported by the comparison with the radiologist’s performance on the same data. On the other hand, we wish to highlight that site 3 had the greatest difference in terms of MRI scanner from site 1, as it had both another vendor and higher field strength. Both these factors could also have contributed in varying degrees to the difference in model performance. Overall, the accuracy on the site 3 external test set can still be considered satisfactory, especially taking into account that it was still not significantly different from that of an expert radiologist (*p* = 0.39).

It is interesting to note that the AUCs obtained in the external test sets by our model (0.73–0.80) are not far from those reported for experienced radiologists interpreting MR images using the EPE grade (0.77–0.81) [9, 10]. The EPE grade has shown a substantial inter- and intra-reader agreement and appears relatively simple to implement being based on relatively few imaging features [10]. However, it is still awaiting prospective validation and requires a certain degree of expertise to be used. It also does not solve the current limitations of mpMRI for EPE detection [36]. Our model exclusively requires lesion segmentation (a step that could also be automated) and would be easy to implement. It can be hypothesized that including our radiomics signature in the EPE grade scoring system might possibly further increase its diagnostic accuracy and reliability while supporting less experienced readers in the EPE assessment. On a similar note, future investigations could assess whether the inclusion of clinical and laboratory data, such as patient age, PSA/PSA-derived biomarkers, or biopsy Gleason score, may further improve our results.

Our study has some limitations that should be acknowledged. Its design was retrospective, which did not allow us to investigate more possible sources of limited radiomics feature reproducibility (e.g., scanner differences) in addition to manual segmentation. However, we believe a retrospective multicenter study is a necessary step after single-center experiences and prior to prospective clinical trials. We had an experienced radiologist from each study center assess the center’s exams, which could determine some bias due to differences in performance. However, this choice was in our estimation better than having a single radiologist read exams acquired on MRI scanners with which he/she may not have been familiar, which could also have negatively influenced the outcome.

In conclusion, the combination of radiomics and ML has confirmed their promising performance for PCa EPE detection even in a multicenter setting. This tool could aid in improving patient management and be a valid support for radiologists in PCa staging. The next step in its development should be a prospective clinical trial.

## Supplementary information

ESM 1 (DOCX 28 kb)

ESM 2 (MODEL 199 kb)

## Abbreviations

ADC: Apparent diffusion coefficient
AUC: Area under the Receiver Operating Characteristics curve
AUPRC: Area under the Precision-Recall curve
DWI: Diffusion-weighted images
EPE: Extraprostatic extension of disease
ICC: Intercorrelation coefficient
ISUP: International Society of Urological Pathology
MCC: Matthew’s correlation coefficient
mpMRI: Multiparametric MRI
PCa: Prostate cancer
PI-RADS: Prostate Imaging and Reporting Data System
PSA: Prostate-specific antigen
ROC: Receiver operating characteristics
SVM: Support vector machine
T2w: T2-weighted
