# Supplementary material for: MRI index lesion radiomics and machine learning for detection of extraprostatic extension of disease: a multicenter study
Source: Eur Radiol. 2021 Apr 1;31(10):7575–83. doi: 10.1007/s00330-021-07856-3 (PMC8452573; doi:10.1007/s00330-021-07856-3)
Supplement: Supplementary file 1 — (DOCX 28 kb) [file 330_2021_7856_MOESM1_ESM.docx]

**Supplementary materials**

*Technical parameters of the Site 1 acquisition protocol (1.5 T scanner, Achieva, Philips Medical Systems, Eindhoven, The Netherlands):*

- Sagittal, axial and coronal TSE T2-weighted sequences: TR 5000 ms, TE 120 ms, slice thickness 3 mm (without spacing), matrix 256x204, FOV 180x180
- Axial diffusion weighted imaging sequence (ss-EPI-SPAIR): TR 2586 ms, TE 78 ms, slice thickness 3 mm (without spacing), matrix 64x63, FOV 180x180, b values = 50, 1000, 1700 s/mm2
- Axial perfusion dynamic contrast enhanced sequence (3D T1-weighted GRE fat suppressed): TR 4,7 ms, TE 2,3 ms, slice thickness 3 mm, matrix 100x100, FOV 180x180, temporal resolution 7 s, total acquisition time 240 s.

*Technical parameters of the Site 2 acquisition protocol (1.5 T scanner, Ingenia, Philips Medical Systems, Eindhoven, The Netherlands):*

- Sagittal, axial and coronal TSE T2-weighted sequences: TR 3356 ms, TE 120 ms, slice thickness 3 mm (without spacing), matrix 188x183, FOV 140x140
- Axial diffusion weighted imaging sequence (ss-EPI-SPAIR): TR 4497 ms, TE 78 ms, slice thickness 3 mm (without spacing), matrix 100x100, FOV 250x250, b values = 0, 500, 1000, 1500, 2000 s/mm2
- Axial perfusion dynamic contrast enhanced sequence (3D T1-weighted GRE fat suppressed): TR 4,9 ms, TE 2,3 ms, slice thickness 3 mm, matrix 150x150, FOV 240x240, temporal resolution 8 s, total acquisition time 240 s.

*Technical parameters of the Site 3 acquisition protocol (3 T scanner, Magnetom Trio, Siemens Medical Solutions, Erlangen, Germany):*

- Sagittal, axial and coronal TSE T2-weighted sequences: TR 4000 ms, TE 101 ms, slice thickness 3 mm (without spacing), matrix 310x320, FOV 200x200
- Axial diffusion weighted imaging sequence (ss-EPI-SPAIR): TR 4900 ms, TE 89 ms, slice thickness 3 mm (without spacing), matrix 144x144, FOV 200x200, b values = 50, 800, 1500 s/mm2
- Axial perfusion dynamic contrast enhanced sequence (3D T1-weighted GRE fat suppressed): TR 4,38 ms, TE 1,55 ms, slice thickness 3 mm, matrix 115x192, FOV 260x260, temporal resolution 6 s, total acquisition time 240 s.

**ICC Segmentations**

Number of slices included in the 3D ROIs annotated on T2-weighted images and ADC maps by each reader for all cases. These were used to calculate feature ICC values and include exclusively those that did not show limited reproducibility due to the manual segmentation process, as detailed in the main text.

| **Lesion_ID** | **T2-weighted Images** | | | **ADC Maps** | | |
| --- | --- | --- | --- | --- | --- | --- |
|  | *Reader 1* | *Reader 2* | *Reader 3* | *Reader 1* | *Reader 2* | *Reader 3* |
| ID_1 | 5 | 5 | 4 | 7 | 5 | 5 |
| ID_2 | 7 | 7 | 7 | 7 | 9 | 9 |
| ID_3 | 4 | 3 | 3 | 3 | 4 | 4 |
| ID_4 | 3 | 3 | 3 | 3 | 4 | 4 |
| ID_5 | 3 | 3 | 3 | 6 | 3 | 3 |
| ID_6 | 5 | 2 | 2 | 3 | 3 | 3 |
| ID_7 | 3 | 4 | 3 | 3 | 3 | 3 |
| ID_8 | 3 | 4 | 4 | 3 | 5 | 5 |
| ID_9 | 2 | 3 | 3 | 2 | 4 | 4 |
| ID_10 | 4 | 5 | 5 | 5 | 4 | 4 |
| ID_11 | 3 | 3 | 3 | 4 | 4 | 4 |
| ID_12 | 5 | 6 | 5 | 5 | 6 | 5 |
| ID_13 | 3 | 5 | 5 | 3 | 6 | 5 |
| ID_14 | 4 | 3 | 3 | 3 | 4 | 3 |
| ID_15 | 8 | 9 | 9 | 8 | 13 | 13 |
| ID_16 | 5 | 5 | 5 | 8 | 8 | 8 |
| ID_17 | 5 | 4 | 4 | 7 | 6 | 6 |
| ID_18 | 4 | 3 | 3 | 3 | 5 | 4 |
| ID_19 | 4 | 2 | 2 | 5 | 4 | 3 |
| ID_20 | 3 | 3 | 3 | 3 | 2 | 1 |
| ID_21 | 3 | 4 | 4 | 3 | 4 | 4 |
| ID_22 | 4 | 4 | 3 | 3 | 4 | 3 |
| ID_23 | 3 | 3 | 3 | 4 | 4 | 4 |
| ID_24 | 1 | 2 | 1 | 1 | 3 | 1 |
| ID_25 | 4 | 3 | 2 | 4 | 4 | 4 |
| ID_26 | 2 | 2 | 2 | 2 | 2 | 2 |
| ID_27 | 4 | 3 | 3 | 2 | 4 | 3 |
| ID_28 | 2 | 3 | 2 | 2 | 5 | 5 |
| ID_29 | 3 | 3 | 2 | 2 | 2 | 2 |
| ID_30 | 2 | 3 | 3 | 2 | 3 | 2 |

**Radiomic features**

1. t2_original_glszSizeZoneNonUniformityNormalized
2. t2_log-sigma-2-0-mm-3D_glcClusterShade
3. t2_log-sigma-4-0-mm-3D_glszSizeZoneNonUniformityNormalized
4. t2_log-sigma-4-0-mm-3D_gldSmallDependenceEmphasis
5. t2_wavelet-LHH_glcCorrelation
6. t2_wavelet-HHL_glcImc2
7. t2_wavelet-HHL_gldLargeDependenceLowGrayLevelEmphasis
8. adc_original_gldLargeDependenceLowGrayLevelEmphasis
9. adc_log-sigma-2-0-mm-3D_glcIdn
10. adc_log-sigma-2-0-mm-3D_gldLargeDependenceLowGrayLevelEmphasis
11. adc_log-sigma-4-0-mm-3D_firstorder_Maximum
12. adc_wavelet-LHL_glszGrayLevelVariance
13. adc_wavelet-HLH_glrlLongRunEmphasis
14. adc_wavelet-HHH_glrlLongRunLowGrayLevelEmphasis

**Radiologist Confusion Matrices**

| Site 2 | | Ground truth | |
| --- | --- | --- | --- |
|  |  | No EPE | EPE |
| Radiologist | No EPE | 20 | 4 |
|  | EPE | 4 | 15 |

| Site 3 | | Ground truth | |
| --- | --- | --- | --- |
|  |  | No EPE | EPE |
| Radiologist | No EPE | 22 | 3 |
|  | EPE | 5 | 16 |

EPE = extraprostatic extension of disease
